# Toxicity and immunogenicity concerns related to PEGylated-micelle carrier systems: a review

**DOI:** 10.1080/14686996.2019.1590126

**Published:** 2019-04-15

**Authors:** Kouichi Shiraishi, Masayuki Yokoyama

**Affiliations:** Division of Medical Engineering, Research Center for Medical Sciences, The Jikei University School of Medicine, Kashiwa, Chiba, Japan

**Keywords:** Polymeric-micelle carrier systems, poly(ethylene glycol) (PEG), toxicity, immunogenicity, anti-PEG IgM, 30 Bio-inspired and biomedical materials, 101 Self-assembly / Self-organized materials

## Abstract

Polymeric-micelle carrier systems have emerged as a novel drug-carrier system and have been actively studied for anticancer drug targeting. In contrast, toxicological and immunological concerns related to not only polymeric-micelle carrier systems, but also other nanocarrier systems, have received little attention owing to researchers’ focus on therapeutic effects. However, in recent clinical contexts, biopharmaceuticals’ effects on immune responses have come to light, requiring that researchers substantively explore the potential negative side effects of nanocarrier systems and of therapeutic proteins in order to develop nanocarrier systems suitable for clinical use. The present review describes current insights into both toxicological and immunological issues regarding polymeric-micelle carrier systems. The review focuses on immunogenicity issues of polymeric-micelle carrier systems possessing poly(ethylene glycol) (PEG). We conclude that PEG-related immunogenicity is deeply related to characteristics of a counterpart block of PEG-conjugates, and we propose future directions for addressing this unresolved issue.

## Graphical abstract

**Figure:**
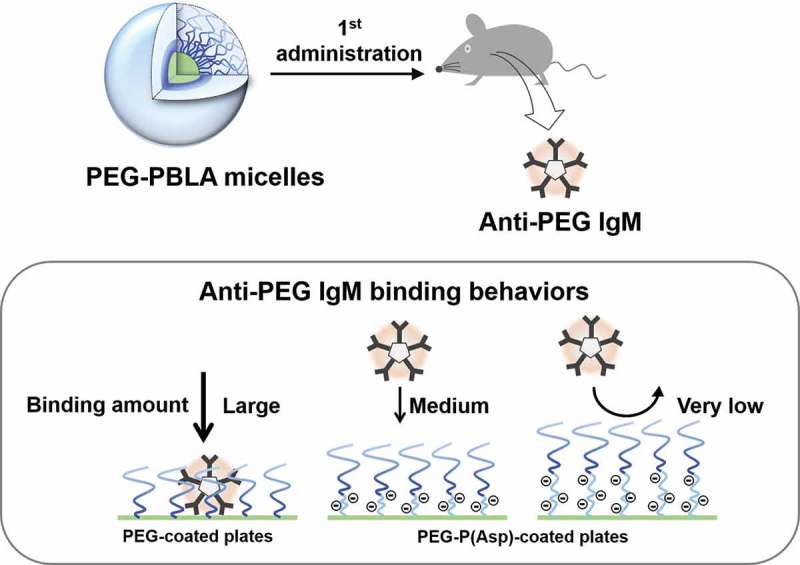

## 1. Introduction

In 1984, Bader and Ringsdorf [1] proposed polymeric micelles formed from block copolymers as a new drug carrier system. Although their study was limited to *in vitro* use of polymeric-micelle structures for the sustained release of drugs, the priority and originality of this study became much more evident with subsequent significant developments in polymeric-micelle research. In the 1980s, two other research groups conducted polymeric-micelle studies with the distinct intention of establishing viable *in vivo* drug-targeting delivery systems. Since the 1990s, more and more research activities have been carried out not only for drug delivery but also for contrast-agent delivery with polymeric-micelle carriers.

Research on polymeric-micelle carrier systems has centered on anticancer drugs [2,3]. Research on the toxicity of polymeric-micelle carriers is very limited, however, mainly because the toxicity of untargeted drugs tends to be much more serious than that of the carriers in anticancer drug targeting. Another reason for this limited research is that carrier toxicity is studied most extensively in pre-clinical stages, resulting in data unavailable to the public. In addition to toxicity, examinations of carriers’ immunological properties are important in clinical applications, since multiple doses are common in clinical settings. If carrier systems induce immunological responses of patients, these responses may inhibit targeting in the second or later doses through production of antibodies specific to the carrier systems. However, these antibody responses are not important issues for anticancer drug-targeting cases because most anticancer drugs suppress the antibody responses. In fact, an immunological response called ‘accelerated blood clearance (ABC) phenomenon’ was not observed [4] in cancer chemotherapy with a PEG-liposomal carrier. In this review, we cover recent researches addressing toxicity and the immunological issues of polymeric-micelle carriers and provide perspectives on material science and technologies for future nanomedicines.

## 2. Toxicity of PEGylated polymeric micelles

Our previous toxicity study [5] was on polymeric micelles formed from poly(ethylene glycol)-*b*-poly(aspartate) (PEG-P(Asp(Bzl))) block copolymers. The poly(aspartate) block was composed of a hydrophilic aspartic acid unit and a hydrophobic benzyl aspartate unit. The ratio of aspartic acid to benzyl aspartate was 11:89. This block copolymer was applied to carriers of camptothecin [6,7] and synthetic retinoids [8,9] such as all-trans retinoic acid. We studied the related toxicities by conducting pathological examinations using Donryu strain rats [5]. We injected them intravenously five times with either 20 mg/kg (low dose) or 200 mg/kg (high dose), every other day for the low dose and every day for the high dose. On sacrifice, we measured body-weight change and the weights of major organs (the brain, heart, lungs, liver, spleen, and kidneys); and after sacrifice on day 30 for the low dose and on day 8 for the high dose, we conducted histological examinations of the brain, thymus, lymph node, heart, lungs, liver, spleen, gastro-intestines, pancreas, kidneys, adrenal gland, ovaries, uterus, muscle, and bone. At each of the two doses, we found no pathological abnormality in any of the examined organs and tissues. We, however, observed an increased number of foamy cells in the lungs and lymph nodes for some micelle-injected rats at the low dose. At the high dose, we observed a significant increase in foamy-cell numbers in the spleen. Then, we carried out immunohistological examinations by using an anti-rat CD68 mouse monoclonal antibody to detect CD68-positive macrophages that are foamy cells. We found marked increases in the CD68-positive macrophages in the spleen, liver, and lungs. By using biotinylated polymeric micelles, we confirmed the accumulation of polymeric micelles in the mononuclear phagocyte system (MPS) cells of the spleen, liver, and lungs. We concluded that micelle administration activated the MPS, and we found no *in vivo* toxicity associated with the MPS activation. This MPS-activation phenomenon seems much less important than the toxic side effects originating from incorporated cytotoxic anticancer-drugs, since the MPS suffered considerable damage from cytotoxic drugs. However, this MPS-related phenomenon may be important if polymeric-micelle carrier systems are applied to delivering drugs that are much less toxic than typical anticancer drugs.

On the other hand, Turecek et al. [10,11] examined toxicological studies of PEG-conjugated (PEGylated) proteins and reported cellular vacuolation for 5 of the 11 approved PEG-protein conjugates and 10 of the 17 PEG-protein conjugates, which are currently in states of early clinical or nonclinical development. The cellular vacuolation was observed typically in the MPS, including the spleen and liver. Cellular vacuolation is the same phenomenon as the MPS activation that we describe above. Turecek et al. also reported that, for some PEG-protein conjugates, cellular vacuolation had been observed in other tissues: namely, lymph nodes, renal tubular cells, synovial cells, salivary glands, testis, melanocytes, the thymus, adrenal glands, the adrenal cortex, the heart, the duodenum, the jejunum, mammary glands, bone marrow, ovaries, the uterus, the cervix, the vagina, adipose tissue, the choroid plexus, and the pituitary gland. (For one PEG-protein conjugate, cellular vacuolation was observed in some—not all—of the above-mentioned tissues.) Among these tissues, those of the choroid plexus, pars nervosa, and pituitary gland attract special attention because they are located near the central nervous system. No adverse effect attributable to the cellular vacuolation was seen by our previous study. Above mentioned our results on polymeric micelles and Turecek et al.’s reports on PEG-conjugated proteins are notable because both PEG-P(Asp(Bzl)) block copolymer micelles and PEGylated proteins possess PEG chains. In our examination of PEG-P(Asp(Bzl)) block copolymer micelles, we observed cellular vacuolation only in the spleen, liver, and lungs; in other words, we observed no vacuolation in other organs or tissues [5]. Currently, we cannot describe relationships between the cellular vacuolation and chemical structures of block copolymer or PEG-conjugates in terms of PEG conjugation. However, we are sure that observation of cellular vacuolation is an important issue for further examinations of PEG-possessing block copolymer micelles, particularly examinations of the choroid plexus and the pituitary gland, both of which are near the central nervous system.

## 3. Immunological issues of PEGylated polymeric micelles

### 3.1. PEGylation and PEG-related immunological issues

As we described above, polymeric-micelle carrier systems have emerged as a novel drug carrier system, and researchers have studied polymeric-micelle carrier systems’ potential as anticancer drug targeting. Extended blood-circulation time periods of nanoparticles, as well as of biopharmaceuticals, are a pre-requisite for efficient therapy (drug targeting) and are frequently achieved by conjugations of hydrophilic polymers, such as poly(ethylene glycol) (PEG). PEG is a synthetic and non-ionic linear polymer, which possesses a repeating unit of ethylene oxide (-CH_2_CH_2_O-) and has been the most widely used polymer for both biopharmaceutics and nanoparticles. PEG-conjugation techniques are called PEGylation [12,13]. The intrinsic characteristics of PEG are chemical inertness, solubility in both water and organic solvents, biocompatibility, and relative non-toxicity.

**Figure 1.**
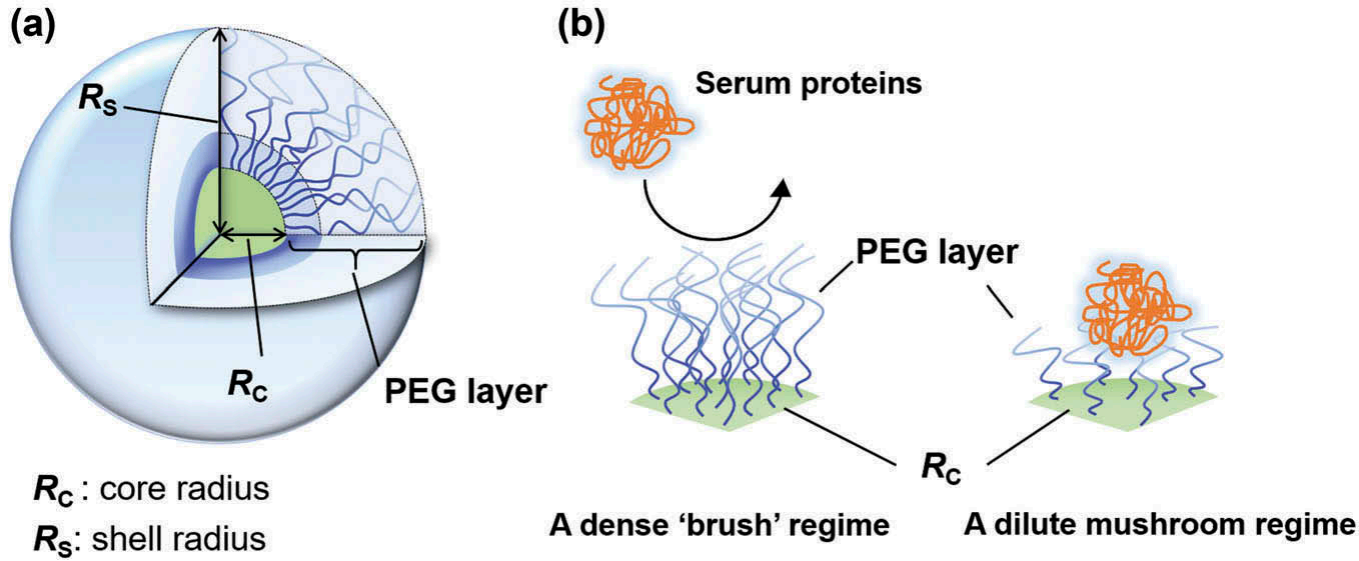
(a) A detail structure of polymeric micelle. (b) A dense PEG brush structure on polymeric micelle surface suppresses adsorption of serum proteins.

PEGs form a hydrated PEG layer, which resists adsorption of serum proteins and phagocytic uptake. This effect has been called a stealth effect. The stealth effect of PEGylation improves the blood circulation half-lives of biopharmaceuticals, as well as nanoparticles. In recent studies, we and other researchers have shown importance of PEGylation density onto nanoparticles’ surface, and they revealed a ‘dense brush’ conformation is required to suppress phagocytic uptake *in vitro* and to exhibit long-blood circulation *in vivo* (Figure 1) [14–18]. These features of PEGylation have led to a new era for developments in biopharmaceuticals and drug carriers. Abuchowski et al. assessed the conjugation of PEG onto proteins, such as bovine serum albumin (BSA) and liver catalase, and claimed that PEGylated BSA improved blood circulation half-lives and elicited antibodies against neither PEG-BSA nor BSA [19]. The results indicate high potency of PEGylation for biopharmaceuticals. PEGylation onto liposomes (PEG-liposomes) exhibited less accumulation in the liver and spleen than did non-PEGylated counterparts [20]. Stealth PEG-liposomes exhibited long blood circulation half-lives. Although PEG-liposomal systems exhibited the stealth effect, the first and only FDA approved PEGylated liposomal drug, CAELYX^TM^/Doxil is a cytotoxic doxorubicin-loaded PEG-liposome system [21].

PEG has been known as a safe, inert, and non-immunogenic synthetic polymer. However, PEG-related immunological issues have received considerable attention [22–28]. Anti-PEG antibodies have been found in patients who were treated with PEGylated nonhuman enzymes [22–25]. Furthermore, circulating anti-PEG antibodies have been found in healthy subjects and are thought to be induced by PEG containing cosmetics and foods [26,28]. In fact, the first study of PEGylation onto BSA exhibited no anti-PEG antibody induction (anti-PEG Abs) and no change in clearance upon repeated injections [19]. The use of PEGylation onto nanoparticles, such as liposomes, micelles, and nanoparticles, has faced PEG-related immunological issues owing to rapid blood clearance of PEGylated nanoparticles (PEG-NPs) upon repeated administration [29–31]. Despite the frequent use of PEGylation to improve half-lives of biopharmaceuticals and nanoparticle carrier systems, research has not elucidated PEG-related immunological issues. Furthermore, researchers have focused almost exclusively on rapid blood clearance of repeatedly administered nanoparticle carrier systems.

If one PEGylated nanoparticle carrier system induces anti-PEG *Ab* responses, repeated administrations of the nanoparticle carrier system exhibits loss of long blood circulation. This phenomenon indicates not only that efficient drug targeting ceases after the second administration, but also that a risk of side effects increases, particularly regarding the high accumulation of both drugs and drug carriers in specific organs. These anti-PEG *Ab* responses may cause serious issues in the drug-targeting field. For example, in previous research, we developed both a polymeric-micelle carrier system for MRI contrast agents (diagnostic purposes) [32] and a polymeric-micelle carrier system for anticancer drugs (therapeutic purposes) [3]. Theranostic treatment, which is a concept for efficient therapeutic treatments in combination with diagnostic treatments, can involve two types of polymeric-micelle carrier systems: one for cancer diagnosis, and the other for cancer therapy. If the polymeric-micelle-carrying MRI contrast agent induces anti-PEG *Ab* responses, the targeting ability of the polymeric-micelle-carrying therapeutic agent will be lost. Furthermore, high accumulation of a cytotoxic agent in specific organs, such as the liver and spleen, may cause serious side effects.

PEG possesses great advantages for the modification of biopharmaceuticals and of nanoparticle carrier systems. More PEGylated biopharmaceuticals and nanoparticle carrier systems are expected to enter the market. To avoid the negative scenarios discussed above, elucidation of PEG-related immune responses is undoubtedly essential for further clinical use of PEGylation. So far, several published reviews have summarized PEG-related immune responses [33–36]. Nevertheless, the collecting of human- and animal-based data may not be enough to elucidate the potent immunogenicity of PEG in present situations. Thus, in this part, we firstly summarize PEG-related immune responses, as well as underlying mechanisms in immune systems. Then, we introduce our approach for anti-PEG responses underlying mechanisms in PEG immunogenicity and discuss why PEGs become immunogenic, with a focus on both the immunogenicity issues of PEGylated micelles and the differences between PEGylated-micelles and PEGylated liposomes. Finally, we introduce recent works to overcome the PEG-related immunological issue.

### 3.2. Induction of humoral immune responses by PEG-nanoparticles

**Figure 2.**
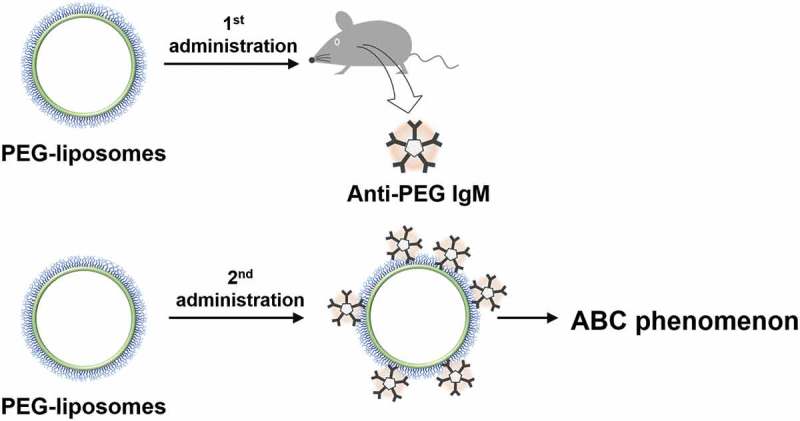
The first PEG-liposome elicited anti-PEG IgM, and the second PEG-liposome exhibited ABC phenomenon.

A first dose of PEG-NPs demonstrates longer blood-circulation time *in vivo* than does a first dose of non-PEGylated counterparts. Like PEGylated proteins, PEGylation onto particle surfaces greatly improves pharmacokinetics. In 1997, Moghimi and Gray reported that a PEG surfactant, poloxamine 908-coated polystyrene particles exhibited rapid clearance upon the second administration [29]. In early 2000, a pioneering work by Dams et al. showed that the first dose of PEG-liposomes exhibited the characteristic of long blood circulation whereas the second dose of PEG-liposomes exhibited rapid clearance with significantly increased accumulation in both the liver and spleen [30,31]. This rapid blood clearance of the second dose of PEG-liposomes has been termed the ‘accelerated blood clearance (ABC) phenomenon’ (Figure 2) [30]. Researchers have only partly identified the factors underlying the ABC phenomenon. The two above-mentioned studies examined pre-treated serum transfusion experiments. Moghimi and Gray failed to induce the ABC phenomenon and, therefore, suggested that enhanced phagocytic activities rather than plasma factors were responsible for the rapid clearance. In contrast, Dams et al. reported that transfusion of both IgG- and IgM-depleted pre-treated serum successfully induced the ABC phenomenon of ^99m^Tc-labeled PEG-liposomes, whereas the transfusion of both the IgG- and IgM-fraction of pre-treated serum induced nothing [30]. Therefore, Dams et al. suggested the possibility that heat-labile 150-kD serum complements are the responsible factor. However, after an anti-rat IgM-sepharose affinity column depleted serum IgM (<20%), residual IgM possibly remained in serum. PEG-liposome-related humoral immune responses have been examined by the Ishida group, which found that anti-PEG IgM antibodies (anti-PEG IgM) play a dominant role in the ABC phenomenon [37]. The group also found that PEG-liposomes elicit anti-PEG IgM after the first administration and that the elicited anti-PEG IgM captures the second administration of PEG-liposomes. The group noted that the presence of anti-PEG IgM correlates with blood clearance of PEG-liposomes and with enhanced accumulation of PEG-liposomes in the liver and spleen.

Researchers have identified the involvement of complement activation in PEG-related immune responses, regarding which Dams et al. suggested some serum factors [38–40]. The complement activation facilitates uptake of PEG-liposomes in phagocytic cells [40]. These results indicate that PEG-liposomes induce anti-PEG IgM responses during the first administration. IgM antibodies are unable to directly facilitate phagocytic uptake owing to the absence of Fc receptors for IgM-mediated phagocytic uptake [41]. However, anti-PEG IgM antibody-bound complexes involve complements, which—in classic pathways during the second administration—facilitate phagocytic uptake by complement receptors on such phagocytic cells as hepatic Kupffer cells, splenic marginal zone and red-pulp macrophages, and blood monocytes [39,42]. Therefore, serum complements are significantly involved in the ABC phenomenon. Although these results have been observed in PEG-liposome cases, and although the roles of complement systems in other PEG-NPs remain unclear, complement systems will likely play an important role in other PEG-NPs. In fact, most nanoparticles have been known to activate complements by either themselves or through serum proteins to some extent. Not only activation of complements, certain complement activation pathways promote anti-inflammatory M2-phenotype macrophages, which further promote tumor growth [43,44]. These suggest possibilities of correlation between nanoparticles, host’s immune system, and tumor tissues. These relations are beyond the scope of this review, however, we must bear in mind that any kind of particles affect host immune systems. Above-mentioned studies have suggested possible roles of anti-PEG Abs, serum factors, and phagocytic activities, all of which are responsible for the rapid clearance phenomenon. So far, several studies have examined various PEGylated or novel hydrophilic polymer-based nanoparticle systems regarding the rapid-clearance phenomenon as it occurs over the course of multiple administrations, and have compared mainly those nanoparticle systems with PEG-liposomes [45–48]. In fact, PEG-liposomes have been used as a positive control for the ABC phenomenon. In other words, PEG-liposomes are the most efficient PEG-NPs to exhibit the ABC phenomenon, and researchers have compared PEG-NPs with PEG-liposomes. It should be mentioned that anti-PEG IgM is necessary for observation of the ABC phenomenon, but does not always ensure the complete absence of nanoparticles in blood (the ABC phenomenon). Previously, we made important findings about the ABC phenomenon. Although PEG-*b*-poly(β-benzyl-L-aspartate) block copolymer micelles (PEG-PBLA micelles) induced anti-PEG IgM, the ABC phenomenon was not observable in PEG-PBLA micelles [49]. This observation is critical to clarifying the ABC phenomenon, which is an integral part of PEG-related humoral immune responses. We will describe this function later in the review. The important point to bear in mind here is that the potency of PEG-NP immunogenicity is a more important issue than the observability or the non-observability of the ABC phenomenon.

The above-mentioned examples refer to PEG-NPs in animal models. In contrast, the first FDA-approved drug, CAELYX^TM^/Doxil®, is a cytotoxic drug involving a doxorubicin-loaded PEG-liposome drug-carrier system. Studies examined the relationship between doxorubicin-loaded PEG-liposomes and anti-PEG Abs and reported that the doxorubicin-loaded PEG-liposomes failed to induce either anti-PEG IgM responses or complement activations [50]. This failure is due to the strong cytotoxic drug’s suppression of the host’s immune system, when the doxorubicin-loaded PEG-liposomes were intravenously administered to mice. In fact, doxorubicin’s strong cytotoxicity effect on the reticuloendothelial system (RES) was associated with improvements in the pharmacokinetics of repeated administrations of Doxil®, indicating that plasma-doxorubicin concentrations of the repeated administrations were higher than the first administration [4]. In contrast, induction of anti-PEG IgM responses during the first administration of a PEG-liposome carrier (without a drug) induced the ABC phenomenon of the doxorubicin-loaded PEG-liposomes. Researchers have known that the first administrations of Doxil® are associated with elevated complements in infusion-related reactions [51,52].

### 3.3. PEGylated micelles and anti-PEG Ab responses

Multiple administrations of PEG-NPs can induce PEG-NPs’ humoral immune responses. Laverman et al. identified the ‘induction’ and ‘effectuation’ phases of the ABC phenomenon [31]. Likewise, we should separately evaluate both induction of anti-PEG *Ab* responses and observation of ABC phenomenon. The presence of anti-PEG Abs is a prerequisite for observing the ABC phenomenon and for observing the decline in therapeutic efficacy. However, elicited anti-PEG Abs do not ensure that we can observe both the ABC phenomenon and the decline in therapeutic efficacy. In the following sections, we describe the induction phase and then the effectuation phase.

#### 3.3.1. The ‘induction’ phase of the antibody response

We previously examined anti-PEG *Ab* responses of two PEG-block copolymer micelles, PEG-PBLA micelles and PEG-*b*-poly(L-lysine-DOTA-Gd) block copolymer micelles (Gd-micelles) [45]. These two PEGylated micelles differed from each other regarding their PEGs’ counterpart block and were diametrically opposed to each other regarding their antibody responses. A single administration of PEG-PBLA micelles exhibited anti-PEG IgM responses, and PEG-PBLA micelle-elicited anti-PEG IgM exhibited cross reactivity to various PEG-conjugates, such as PEG-PBLA, PEG-DSPE, and PEG-PLA. In fact, PEG-PBLA micelle-elicited anti-PEG IgM induced the ABC phenomenon of PEG-liposomes. In contrast to PEG-PBLA micelles, Gd-micelles exhibited no anti-PEG IgM response, although we examined several doses for the first administration. Furthermore, we confirmed that a single administration of 500 kD molecular weight of PEG did not induce the ABC phenomenon [45]. Some other study also revealed that a single administration of certain chain lengths of free PEGs did not induce anti-PEG *Ab* responses [53]. These studies indicate that PEGs have a haptogenic property and that PEG-conjugates induce anti-PEG *Ab* responses. This haptogenic property of PEGs strongly complements the immunogenicity of conjugated proteins or NPs. Our aforementioned examples indicate that a PEG-conjugate possessing a hydrophilic counterpart block has extremely weak immunogenicity (not strong immunogenicity). Therefore, we conclude that an intact PEG is indeed not immunogenic by itself, but is haptogenic. We use the term ‘intact PEG’ to refer to the repeating ethylene oxide unit (-(CH_2_CH_2_O)*_n_*-). Intact PEG can become immunogenic when PEG is bound to either an immunogenic protein or a lipid counterpart block, and our results indicate that a hydrophobic counterpart block plays a significant role in anti-PEG IgM responses. This PEG’s haptogenic property is most likely the reason for which PEGylated nonhuman enzymes, such as uricase and asparaginase, exhibited the most severe responses, whereas other protein cases exhibited no responses [22–24]. In summary, induction of PEG-related antibody responses is deeply related to the characteristics of the counterpart blocks of PEG-conjugates.

#### 3.3.2. The ‘effectuation’ phase of the antibody response

We have shown the advantages of PEGylated micelles against PEG-liposomes and have found, specifically, that both PEG-PBLA micelles and Gd-micelles do not exhibit the ABC phenomenon [45,49], whereas PEG-liposomes do. From this point, two important questions arise: First, why do PEG-liposomes normally exhibit the ABC phenomenon? And second, how does elicited anti-PEG IgM in blood affect the second dose of PEG-NPs? To answer the first question, we examined the second ‘effectuation’ phase of the ABC phenomenon by using both PEG-PBLA micelles and PEG-liposomes [54].

**Figure 3.**
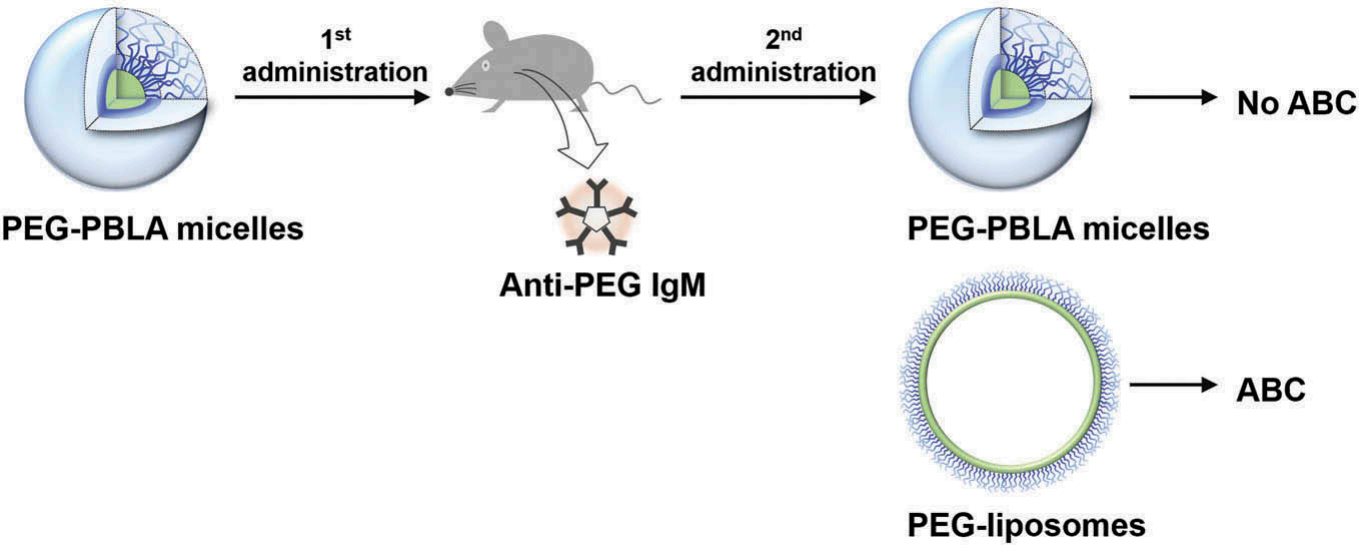
PEG-PBLA micelles induced anti-PEG IgM responses in mice, however, PEG-PBLA micelles exhibited no ABC phenomenon.

In our previous study, we reported a significant difference between PEG-liposomes and PEG-PBLA micelles in the second phase of the ABC phenomenon [54]. The two carriers induced anti-PEG IgM responses, and elicited anti-PEG IgMs were cross reactive to both PEG-liposomes (PEG-DPSE) and PEG-PBLA. However, only PEG-liposomes exhibited the ABC phenomenon (rapid blood clearance), whereas the same PEG mole dose of PEG-PBLA micelles did not exhibit the ABC phenomenon (Table 1). Very interestingly, PEG-PBLA micelles did not exhibit the ABC phenomenon, whereas PEG-PBLA micelles induced anti-PEG IgM responses (Figure 3).

**Table 1.** Summary of characteristic differences between PEG-liposomes and PEG-PBLA micelles.

1^st^ injection	Size/nm	Anti-PEG IgM induction	2^nd^ injection	ABC phenomenon	*K*_d_/M*	PEG nmol/mouse	Particles number *N*/mouse
PEG-liposomes	130	Yes	PEG-liposomes	Yes	1.0 × 10^−7^	5.1	2.1 × 10^11^
			PEG-PBLA micelles	No			
PEG-PBLA micelles	90	Yes	PEG-liposomes	Yes	1.4 × 10^−7^	4.5	1.6 × 10^12^
			PEG-PBLA micelles	No			

* We estimated *K*_d_ values of anti-PEG IgM to PEGylated NPs by means of ELISA. *K*_d_ values obtained a concentration > 20μg/mL.

This fact motivated us to examine behaviors of anti-PEG IgM. One possible explanation for the difference between the two types of carriers is a difference between the anti-PEG IgM’s binding affinity to the PEG-liposomes and the PEG-PBLA micelles. We expected that the binding affinity of anti-PEG IgM to PEG-liposomes would be greater than that of anti-PEG IgM to PEG-PBLA micelles. Therefore, we examined binding behaviors of anti-PEG IgM to the two types of carriers by means of an inhibition enzyme-linked immunosorbent assay (ELISA). However, we found that two types of carriers’ binding behaviors to anti-PEG IgM were the same at approximately identical PEG mole concentrations. This finding indicates that anti-PEG IgM exhibited nearly the same binding affinity to the PEG of each type of carrier.

We also noticed that no anti-PEG IgM remained in plasma after the second administration of PEG-PBLA micelles. This finding indicates that injected PEG-PBLA micelles completely consumed anti-PEG IgM antibodies. In sum, PEG-PBLA micelles induced anti-PEG IgM responses, and the elicited anti-PEG IgM induced the ABC phenomenon for PEG-liposomes. Elicited anti-PEG IgM induced no ABC phenomenon for PEG-PBLA micelles, and no anti-PEG IgM remained in plasma after administration of PEG-PBLA micelles. We concluded that a difference in the number of injected particles is the reason for the difference between PEG-liposomes and PEG-PBLA micelles regarding the ABC phenomenon. We determined the numbers of particles by using the aggregation number of PEG-NPs. In nearly identical PEG-mole doses, a dose of PEG-liposomes included 10 times fewer particles than a dose of PEG-PBLA micelles. This dramatic difference explains why PEG-liposomes easily exhibit the ABC phenomenon, whereas other PEG-NPs do not. In general, most of polymeric micelle particles exhibit a size range between 10 and 100 nm, and the aggregation number of polymeric micelle particles ranged between 10^1^ and 10^3^. In contrast, PEG-liposomes of the 100-nm size needed approximately 10^4^ PEG molecules in each PEG-liposome particle. These considerations can explain the difference between the two types of carriers in the effectuation phase of the ABC phenomenon. In fact, our previous experiments proved that a pre-mixed solution of anti-PEG IgM and PEG-liposomes in the ratio of 10 anti-PEG IgM to one PEG-liposome particle exhibited the ABC phenomenon [54].

These experiments point to an important aspect. An experiment to observe whether or not the ABC phenomenon is present is not a quantitative evaluation. More importantly, to discuss immune responses of PEG-NPs, we need quantitative evaluations, such as evaluations of plasma concentrations of elicited anti-PEG IgM, evaluations of the ratios of injected PEG-NPs to elicited anti-PEG IgM, and evaluations of the number of injected PEG-NPs captured in the presence of elicited anti-PEG IgM.

### 3.4. Underlying mechanisms in immune systems

The first administration of PEG-NPs stimulates responses by host immune systems. Anti-PEG *Ab* responses are induced after the first administration or repeated administrations of PEG-NPs. Therefore, anti-PEG Abs affect the second or later administrations of those PEG-NPs.

In a number of studies, Ishida and his colleagues examined PEG-liposome-related mechanisms. The researchers found that the spleen plays an important role in anti-PEG IgM responses. In the PEG-liposome studies, a part of administered PEG-liposomes accumulated in the splenic marginal zone (MZ), where antigens accumulate for induction of humoral immune responses [55,56]. Three to four days after the first administration of PEG-liposomes, anti-PEG IgM could be found in blood. In a mouse model, serum anti-PEG IgM typically exhibited the highest level at week 1 after the first administration of PEG-liposomes, but the level of anti-PEG IgM depended on a dose of PEG-liposomes.

The aforementioned researchers further examined anti-PEG IgM responses in T cell-deficient BALB/c nude mice, and those mice exhibited anti-PEG IgM responses [57,58]. In contrast, splenectomized mice prior to or immediately following the first PEG-liposome administration exhibited decreases in anti-PEG IgM responses, whereas the splenectomized mice exhibited rapid clearance of PEG-liposomes four or more days after the first administration. Furthermore, the researchers examined both severe combined immunodeficient (SCID) mice (B and T cell-deficient) and nude mice (T cell-deficient) and found that SCID mice exhibited no anti-PEG IgM responses [59]. Therefore, the researchers concluded that the PEG-liposomes elicited T cell-independent (TI) IgM responses. So far, researchers have identified only no or very weak IgG responses against PEG (anti-PEG IgG) in relation to PEG-NPs without encapsulated drugs.

We have discussed the classic pathway of complement activations by IgM-bound PEG-liposomes complex in chapter 3.2. On the other hand, it has been known that not only administration of CAELYX^TM^/Doxil®, but also administration of other nanoparticles (micelles, liposomes, nanospheres, and others) involves complement activation through serum protein absorptions [60–63]. Complement is an ancient component of innate immunity that plays significant roles in host innate immunity, and more than 30 complements are known. Complements have been activated either directly or indirectly by three different pathways: classical, alternative, and lectin pathways. For example, absorption of serum proteins on certain nanoparticles induces conformational changes of the serum proteins, and complements are activated through the serum proteins [64–67]. Complement activation via the lectin pathway or the alternative pathway is confirmed in patients who receive the first administration of Doxil® [51,52]. In one study, severe hypersensitivity reactions to Doxil® were found in 25% of Doxil®-treated patients [4], and Doxil caused complement activation in 72% of Doxil®-treated patients. Furthermore, *in vitro* studies for evaluation of complement activation indicated that PEG-liposomes without a cytotoxic doxorubicin were able to activate complements [51,52]. Activation of specific complements release further complement products. Like the third complement product (C3), C3 is activated through the alternative pathway, and further release C3b and iC3b by enzymatic reactions, on the other hand, further complement activation indirectly induced C3a and C5a, which are known as anaphyatoxins [39,68]. Since anaphylactic or hypersensitivity reactions by the 1^st^ administration of Doxil have been observed in patients, complement activations are deeply related to anaphylactic reactions. Therefore, we must keep in mind possibility of complement activations to some extent. Further studies of complement activation in relation to PEG-nanoparticles merit rigorous evaluation for immune-safe-materials.

### 3.5. Underlying mechanisms in PEG immunogenicity and an evaluation of PEG-related epitopes

We examined the binding affinity of anti-PEG IgM to PEG-liposomes and polymeric micelles and found that the binding affinity was a counterpart block of PEG-conjugates’ hydrophobicity-dependence [54]. In the study, we revealed that the binding affinity of the anti-PEG IgM to a PEG-conjugate possessing weak hydrophobicity is low, whereas the binding affinity of the anti-PEG IgM to a PEG-conjugate possessing strong hydrophobicity is high. As a result, PEG-conjugates exhibiting high affinity to anti-PEG IgM need very low concentrations to inhibit bindings, whereas PEG-conjugates exhibiting low affinity to anti-PEG IgM need high concentrations to inhibit bindings. In fact, Sherman M. R. et al. suggested the advantages of hydroxyl-PEGylation over methoxy-PEGylation [69]. The aforementioned study includes two important facts. First, free PEGs can inhibit anti-PEG *Ab* bindings, but need to be at very high concentrations for the inhibition to manifest itself. Second, the researchers and others found that the methoxy group’s role in bindings was weak, but greater than the terminal hydroxyl group’s corresponding role [70,71]; specifically, intact PEG played a very weak role in bindings. In sum, the various cited studies indicate that any hydrophobic parts of PEG-conjugates, even a terminal very weak hydrophobic methoxy group, play an important role in anti-PEG *Ab* bindings.

Therefore, the aforementioned Sherman’s *in vitro* study regarding free PEGs’ inhibition of anti-PEG bindings and the aforementioned *in vivo* study regarding free PEGs’ failure to induce anti-PEG IgM responses do not contradict each other. Results indicate that PEG-specific antibodies exhibit very weak binding to intact PEGs but that PEG-specific antibodies exhibit greater binding to PEG-conjugates, which possess strong affinity at either terminals. We, along with many researchers, have observed PEG-specific antibody responses, but an important question remains unanswered: what is the exact epitope for PEG-specific antibodies?

**Figure 4.**
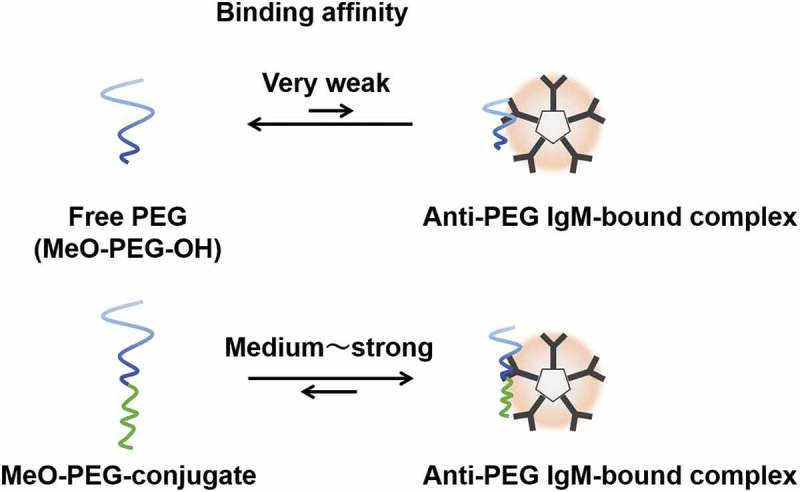
Schematic images of equilibrium between an anti-PEG IgM-bound complex and (a) free PEG (MeO-PEG-PH) and (b) PEG-conjugate (MeO-PEG-conjugate). Binding affinity of MeO-PEG conjugate is a counterpart block of PEG-conjugates’ hydrophobicity-dependent.

We have suggested that PEG molecules can access anti-PEG IgMs, which possess a specific site for PEG molecules. For one example, a MeO-PEG-OH (an example of free PEGs, where MeO stands for a methoxy group) possessing no significant cohesive force exhibits an equilibrium between a MeO-PEG-OH and a bound complex of anti-PEG IgM-PEG (Figure 4(a)). But without cohesive forces for forming the two molecules’ complexes, the equilibrium shifts to the left (free MeO-PEG-OH). By contrast, PEG-conjugates possessing strong cohesive force easily form a bound complex of anti-PEG IgM-PEG-conjugates, which appears to the right of the equilibrium (Figure 4(b)).

**Figure 5.**
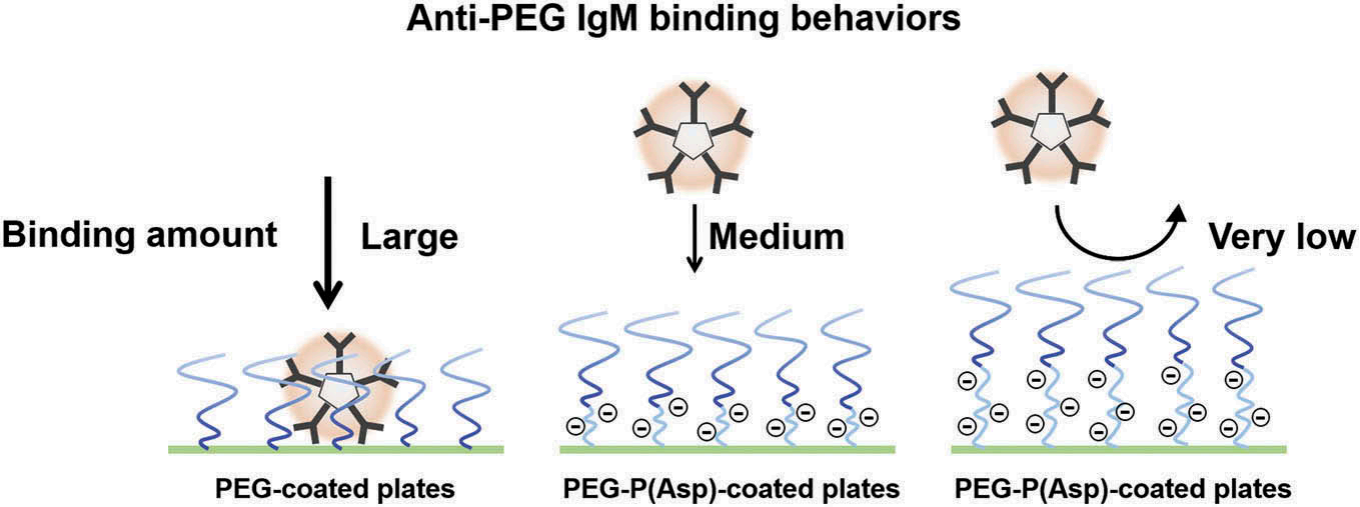
PEG-conjugate possessing P(Asp) units between a PEG and a hydrophobic block suppressed bindings of anti-PEG IgM in a P(Asp) ‘chain length’-dependent manner.

Although most relevant ELISA studies have used a simple ELISA method for anti-PEG *Ab* detection, those experiments have never referred to actual coated amounts of PEG-conjugates. Because free PEGs do not possess strong cohesive force, coating of free PEGs is not possible. Instead, researchers have used PEG-conjugates. Elicited anti-PEG IgM exhibited cross reactivity to most PEG-coated plates. In contrast, each counter block of PEG-conjugates possesses such characteristics as cohesive force, electric charges, and steric hindrance. These PEG-conjugates’ characteristics may affect anti-PEG IgM’s bindings to PEG-coated plates. Therefore, we must keep in mind that comparisons of anti-PEG IgM’s bindings to two PEG-conjugates are qualitative evaluations, although we have observed the cross reactivity of anti-PEG IgM to PEG-coated plates. Schellekens et al. pointed out the necessity of precise assays for anti-PEG *Ab* detection to quantify anti-PEG *Ab* responses [33]. Therefore, researchers have developed novel methods to detect anti-PEG antibodies in different ways [72–76]. For example, Zhang et al. have presented an assay for anti-PEG antibody by means of a surface plasmon resonance (SPR) biosensor technique [72]. Aćimović et al. have developed a method to elucidate anti-PEG antibody-antigen interaction dynamics by means of localized SPRs (LSPRs) [76]. The LSPR sensing technique is able to elucidate single-molecule equilibrium fluctuations between PEG molecules on gold nanorods and an anti-PEG antibody molecule. To elucidate binding behaviors of anti-PEG IgM, we also examined anti-PEG IgM bindings by the use of different types of PEG-conjugates. We used a PEG-PBLA possessing a methoxy terminal for coating (molecular weight of PEG = 12,000). In addition, we synthesized two new triblock copolymers, PEG-*b*-poly(aspartic acid)-*b*-poly(L-phenylalanine) triblock copolymers (PEG-P(Asp)-P(Phe)), which possess two different chain lengths of polyanionic P(Asp) blocks between a PEG chain and a hydrophobic block, and used for coating [54]. In the aforementioned report, we evaluated actual coated amounts of PEG-block copolymers by the use of fluorescent probe-labeled PEG-block copolymers, and the three polymers did not significantly differ from one another regarding each one’s coating amount. In an ELISA experiment, we first confirmed the cross reactivity of anti-PEG IgM, and anti-PEG IgM bound to both PEG-PBLA-coated plates and PEG-P(Phe)-coated plates. All three polymers possessed a methoxy terminal and the same molecular weight of PEG. However, insertion of a P(Asp) block between the PEG and hydrophobic conjugates suppressed anti-PEG IgM bindings; namely, anti-PEG IgM bindings formed in a P(Asp) ‘chain length’-dependent manner (Figure 5). This ELISA study points to two intriguing findings: anti-PEG IgM does not strongly bind to MeO-PEG part, but does strongly bind to PEG-hydrophobic conjugates, where hydrophobic parts are positioned within the proximity of a PEG chain. Possible roles of P(Asp) chains include establishing a significant physical distance between a PEG chain and a hydrophobic surface and establishing polyanionic environments that may cause electrostatic repulsion. The exact epitope for PEG is still unknown. However, this ELISA study indicates very weak specific interactions between intact PEG chains and anti-PEG IgM. This scenario strongly supports the assertion that PEGs have haptogenic properties. Furthermore, this characteristic of the intact PEG leads us to consider relationship between induction of PEG-specific antibodies and characteristics of PEG-conjugates. Firstly, the induction step of antibody responses is considered to be bindings of PEG-conjugates on surface PEG-specific IgM/IgD receptors. Therefore, the induction of anti-PEG IgM by PEGylated nanoparticles will be affected by conjugate blocks of PEG-conjugates. Secondary, if PEG-specific antibodies have been induced through the intact PEG chain possessing very weak cohesive forces, it can be easily imagined that induced antibodies are specific not only to PEG chains, but also to other polymers. In fact, liposome-injected mice exhibited rapid clearance of the second injection of PEG-liposomes [31,77]. Furthermore, there are direct examples that anti-PEG antibodies have shown cross-reactivity to other polymers/foreign antigens [78,79]. Therefore, PEG-specific immunogenicity indicates broad, and diversity of PEG-specific antibodies.

### 3.6. Conceptions of anti-PEG immune responses and future directions

PEGylation reduces the immunogenicity of non-human proteins and has become a promising technique to improve the pharmacokinetics of biopharmaceuticals. However, growing evidence indicates the potent immunogenicity of both PEG-NPs and PEGylated biopharmaceuticals.

Therefore, the research community should develop strategies to overcome PEG-related immune responses. Researchers have examined the use of alternative hydrophilic polymers, instead of PEG, to overcome PEG’s immunological issues [44–46,80–84]. Researchers suggested that these polymers form self-assembled nanoparticles and exhibit less potent immunogenicity than do PEG; more specifically, alternative hydrophilic polymers exhibit no ABC phenomenon upon repeated administrations. Therefore, novel hydrophilic polymers, instead of PEG, may have potential as drug-delivery vehicles. On the other hand, the absence of an observable ABC phenomenon does not entail an absence of potent immunogenicity in these hydrophilic polymers. Various synthetic polymers, as well as natural products, have exhibited induction of antibodies. Therefore, researchers should undertake further and precise evaluations pertaining to the use of alternative hydrophilic polymers. We should keep in mind that a specific antibody against each polymer merits further evaluation, as well. Another approach to overcoming PEG’s immunological issues is the use of immunosuppressive agents or immunosuppressive conditions that help prevent specific PEG-related immune-response pathways and undesirable side effects [85,86]. The method may suppress PEG-specific immune responses. However, the use of the agents can trigger side effects. How we specifically suppress PEG-related immune responses is a critical issue already under examination. In conclusion, complete suppression of PEG-related immune responses remains a formidable challenge and is of significant importance to the future design of drug carriers and biopharmaceuticals.
